# Skin Permeability of Perfluorocarboxylic Acids Using Flow-Through Diffusion on Porcine Skin

**DOI:** 10.3390/toxics12100703

**Published:** 2024-09-27

**Authors:** Andrew Stephen Hall, Ronald Baynes, Laura M. Neumann, Howard I. Maibach, R. Bryan Ormond

**Affiliations:** 1 Textile Protection and Comfort Center, Wilson College of Textiles, North Carolina State University, Raleigh, NC 27606, USA; ashall5@ncsu.edu; 2 Center for Cutaneous Toxicology and Residue Pharmacology, College of Veterinary Medicine, North Carolina State University, Raleigh, NC 27695, USA; ronald_baynes@ncsu.edu (R.B.); lmneuman@ncsu.edu (L.M.N.); 3 Department of Dermatology, University of California, Oakland, CA 94607, USA; howard.maibach@ucsf.edu

**Keywords:** dermal absorption, perfluorocarboxylic acids, flow-through diffusion, porcine skin

## Abstract

Per- and polyfluoroalkyl substances (PFAS) are found in a variety of places including cosmetics, rain jackets, dust, and water. PFAS have also been applied to occupational gear to protect against water and oils. However, PFAS have been identified as immunosuppressants and perfluorooctanoic acid (PFOA), a specific PFAS, has been identified as carcinogenic. Since there is a risk for dermal exposure to these compounds, there is a need to characterize their dermal absorption. Using *in vitro* flow-through diffusion, skin permeabilities were determined for ^14^C-labeled perfluorooctanoic acid (PFOA), perfluorohexanoic acid (PFHxA), and perfluorobutanoic acid (PFBA) using porcine skin. Tests were conducted over 8 h with either acetone or artificial perspirant as the vehicle. PFBA was found to have greater permeability than PFHxA, likely due to having a smaller molecular weight. The dosing vehicle did not appear to impact permeability rates but impacted the disposition through the skin model. While these PFAS compounds showed a low permeability rate through the skin membranes, they can stay in the skin, acting as a reservoir.

## 1. Introduction

Per- and polyfluoroalkyl substances (PFAS) are compounds that contain at least one C_n_F_2n+1_ moiety and are used in various products including car seats, upholstery, food packaging, and cosmetics [1,2,3]. The carbon‒fluorine bond is one of the strongest bonds which gives PFAS excellent durability. Due to the bond strength, other attractive forces are minimized, making PFAS highly repellent, and therefore PFAS are typically used to impart water and oil repellency to fabrics and other materials.

The downside of excellent durability is that once the useful life of the product is over, PFAS do not break down but remain in the environment. There are thousands of different compounds that can be classified as PFAS but there are not enough resources to determine the toxicity of each compound. Some perfluorocarboxylic acids (PFCA), a subclass of PFAS, alter liver enzymes, increase cholesterol levels, and reduce antibody response [4]. Additionally, perfluorooctanoic acid (PFOA), one of the most studied PFAS, has been classified as a Group 1 carcinogen, carcinogenic to humans [5].

Since PFAS are used in various products and contaminate the environment, there are multiple pathways of exposure. Some PFAS can volatilize and exposure can be via inhalation while PFAS-contaminated water and foods can cause ingestion exposure. While dermal absorption has been assumed to be the least likely pathway for PFAS exposure in comparison to inhalation and ingestion [6,7], it is still a potential source of exposure. Recently, neutral PFAS have been assessed to have greater exposure to skin than inhalation when airborne [8].

While there are significant concerns regarding environmental exposure to PFAS for the public, there is also a greater concern for those exposed to PFAS due to occupational reasons. PFAS are used to impart liquid repellency to materials used in protective clothing to provide protection to the wearer; such fields include medical, firefighting, and the oil and gas industry. While a highly controversial assessment, some groups may identify these uses of PFAS as essential uses, meaning there are no suitable alternatives that are economically or technologically available to provide the same level of performance or protection that may be required by the end user [9]. The wearers of these garments may be dermally exposed to PFAS at a chronic level from consistent wear. Even though protective clothing may be required to ensure the safety against occupational hazards, an understanding of the risk of PFAS exposure via treated textiles is needed.

While dermal absorption of PFAS poses some potential risk, limited information has been provided to determine dermal absorption of PFAS. One *in vivo* study was conducted on a single human subject where a sunscreen formulation dosed with ^13^C-labeled PFOA was applied to the subject’s entire body, and the subject was instructed to not shower for 48 h [3]. For 115 days, blood samples were taken from the subject to measure the plasma concentration. PFOA continued to absorb into the body for 10 days until reaching 118 ng/L [3]. Franko conducted an *in vitro* study using a flow-through diffusion system with mouse and human skin to determine the permeability of PFOA in acetone [10]. In the infinite dose experiment, PFOA was found to have a median permeability coefficient of 4.4 × 10^−5^ cm/h. Along with the permeability data, the study found differences in permeability based on the pH and ionization of PFOA. At a pH below its pKa (~3.8), PFOA was unionized and could more easily pass through skin [10].

Another study used an *in vivo* rat model to determine the absorption of 15 different PFAS compounds, including PFCAs, perfluoroalkyl sulfonates (PFSAs) and fluorotelomer phosphate diesters (diPAPs), for 144 h [11]. The study determined the impact of chemical structure and chain length on dermal absorption. Notably, compounds smaller than 500 Dalton were detected in the blood and urine of the rats. Molecules over 500 Dalton are typically unlikely to pass through the corneal layer [12]. There were peak blood concentrations at three days into the experiment, which began to decrease afterwards.

Ragnarsdóttir et al. has expanded the observation of *in vitro* dermal absorption of PFCAs and PFSAs by using a 3D human skin-equivalent model, an artificial skin tissue that simulates human skin composition [13]. This experiment was conducted using a static cell that was unoccluded using methanol as a vehicle. PFOA was found to have a permeability coefficient of 3.82 × 10^−3^ cm/h, two orders of magnitude higher than found with Franko et al. [10,13]. Compounds smaller than PFOA were found to have increased permeability coefficients.

This study focused on characterizing the dermal absorption of PFOA, perfluorohexanoic acid (PFHxA), and perfluorobutanoic acid (PFBA) using porcine skin, which is known to correlate well with human skin, but has not been previously used to model the dermal absorption of PFAS [14]. The compounds were evaluated using a flow-through diffusion system dosed with acetone as described by Franko et al. [10]. In order to evaluate vehicle effects, artificial perspirant was also used to provide a simulated human exposure in a hot environment. The duration of this study was 8 h to simulate occupational exposure.

## 2. Materials and Methods

Perfluorobutanoic acid [1-^14^C] sodium salt, perfluorohexanoic acid [1-^14^C] sodium salt, and perfluorooctanoic acid [1-^14^C] sodium salt, shown in Table 1, were acquired from American Radiolabeled Chemicals, Inc., in an ethanol solution. Each compound was prepared separately in either acetone (99.3%, J.T. Baker) or artificial eccrine perspiration stabilized with bactericide and fungicide at a pH of 4.5 (Pickering Laboratories, Mountain View, CA, USA). Flow-through cells were 9 mm (0.64 cm^2^) in-line diffusion cells from PermeGear (Hellertown, PA, USA). Collection media were made one day prior to experiments and chemicals are listed in the Appendix A. Pigs used were sourced from the North Carolina State University College of Veterinary Medicine Tissue Sharing Program. Animals had been used for previous research studies that did not involve topical treatments. All animals were female of varying ages and over 20 kg. Skin was harvested after humane euthanasia via pentobarbital overdose given intravenously.

### 2.1. Flow-Through Diffusion Cell Experiment

Flow-through studies were conducted using a method from Bronaugh and Stewert [16]. The study was carried out using fresh porcine skin dermatomed to 400–500 µm with a Padgett Dermatome (Padgett Instruments, Kansas City, MO, USA). The dermatomed skin was then punched into discs (19 mm diameter) and placed into the flow-through diffusion cells, with a dosing surface area of 0.64 cm^2^. The collection media was a solution of bovine serum albumin maintained at a pH of 7.3 to 7.5. The media temperature and flow-through cells were set to 37 °C using a Brinkmann constant temperature circulator. The flow rate of the collection media was set to 4.0 mL/h and collected at 0, 15, 30, 45, 60, 90, 120, 180, 240, 300, 360, and 480 min. The donor vehicle was prepared to achieve approximately 15 µg/cm^2^ topical exposure with either acetone or synthetic eccrine perspiration. Total dosing masses of PFCA compounds were in the range 5–15 µg. Each donor chamber was dosed with 100 µL of dosed vehicle onto the skin disc surface and occluded by capping the donor chamber. For PFOA and PFHxA, ten replicates were dosed for each vehicle while PFBA had six replicates dosed for each vehicle. After the last timepoint, the dosed skin area was swabbed to remove any excess dosing vehicle then swabbed with a solution of water with 1% of Dawn dishwashing liquid (Procter and Gamble). Both swabs were immersed in 10 mL of ethanol to determine what was left on the skin surface. The dosing area was then tape-stripped six times with cellophane tape to determine levels of analyte left in the upper layers of skin in order to capture most of the stratum corneum layers. Tape strips were then extracted with 10 mL of ethyl acetate. Afterwards, 0.64 cm^2^ was cut from the dosed area of each skin disk and dissolved in 2 mL of Biosol (National Diagnostics, Atlanta, GA, USA). The peripheral skin was also dissolved in 2 mL of Biosol. Biosol solutions were then incubated at 50 °C for 8–12 h for radiochemical analysis.

#### 2.1.1. Chemical Analysis

From each specimen of sample type (receptor fluid, swabs, tape strips, skin), 1 mL was transferred to a scintillation vial and 15 mL of BioScint (National Diagnostics, Atlanta, GA, USA) was added. Each vial was then measured using a Tri-Carb 2910 TR scintillation from PerkinElmer to determine total ^14^C. Five to eight replicates of each sample type were collected.

#### 2.1.2. Calculations

To calculate the dose absorbed in the receptor fluid, scintillation detections were measured in disintegrations per minute (dpm) and converted to curies. Using Table 1, curie values were converted to moles and used to determine the mass of sample depending on the compound of interest. The dosing surface area of 0.64 cm^2^ was then used to determine the concentration of dose absorbed.

For this study, absorption is defined as the percentage of the applied dose that was detected in the receptor fluid after the eight-hour exposure. Flux (µg/cm^2^/h) was determined based on the slope of cumulative mass after breakthrough to time (h) curve. Permeability coefficients (cm/h) were determined by the ratio of calculated flux to the average applied dose. A two-way ANOVA was conducted using Prism Graphpad with a 0.05 significance level.

## 3. Results

**Figure 1:**
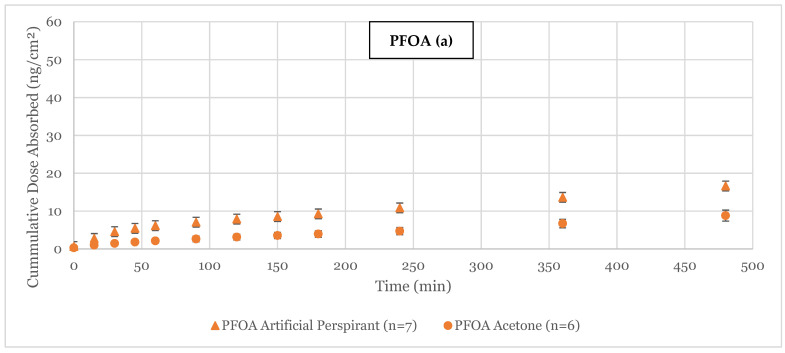

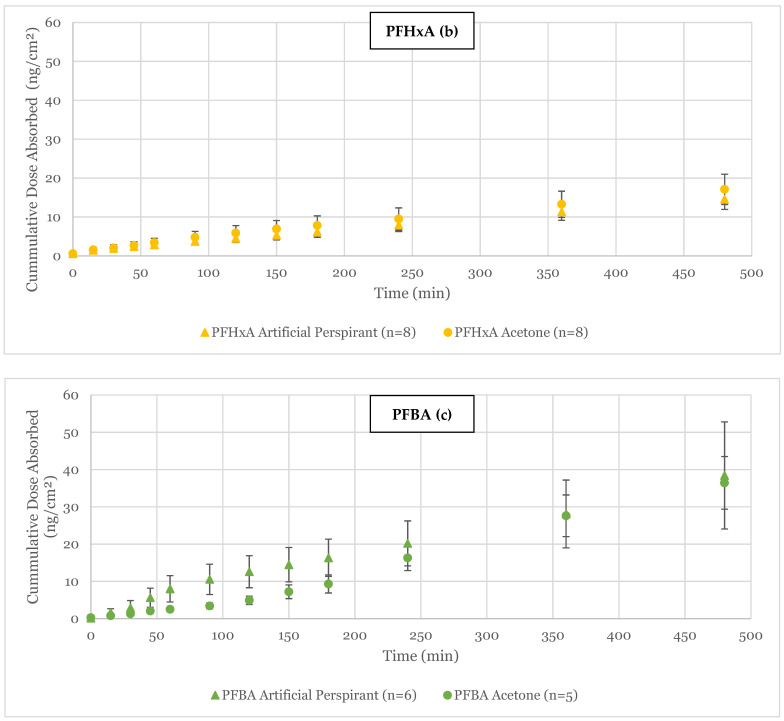
Cumulative absorption (ng/cm^2^ ± standard error) measurements of PFOA (**a**), PFHxA (**b**), and PFBA (**c**) through porcine skin using flow-through with either artificial perspirant or acetone as a vehicle with n replicates.

In the absorption of PFCA compounds, it follows that the larger the compound and higher the LogK_ow_ (octanol-water coefficient), the less compound will be absorbed through skin [11,12,13]. After 8 h, PFBA absorbed the most (38.4 ± 14.4 ng/cm^2^, 36.4 ± 7.1 ng/cm^2^), followed by PFHxA (14.6 ± 2.6 ng/cm^2^, 17.1 ± 3.9 ng/cm^2^) and PFOA (artificial perspirant 16.6 ± 3.2 ng/cm^2^, acetone 8.8 ± 1.5 ng/cm^2^), as shown in Figure 1. Some skin samples had leaked and were not included in analysis. For the amount of dose detected in the perfusate, no significant difference was detected between the dosing vehicles (*p* = 0.6344); however, there was a significant difference between dosing compounds (*p* = 0.0010). For the permeability coefficients (Table 2), the same was true with dosing vehicles having no significant difference (*p* = 0.9967), while there was a significant difference between compounds (*p* = 0.0079).

**Figure 2:**
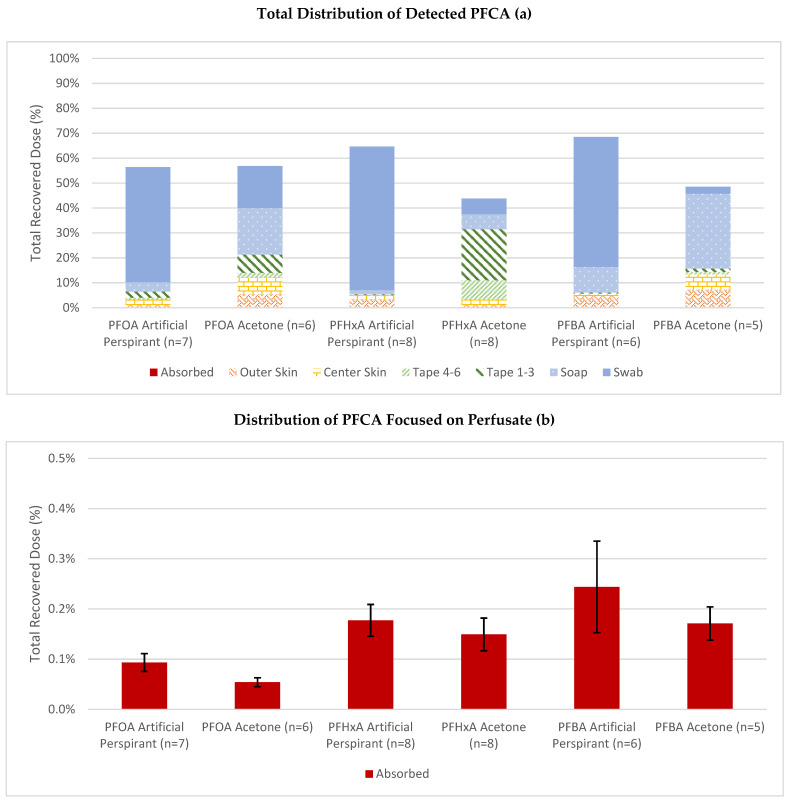
Percentage distribution of PFOA, PFHxA, and PFBA in the flow-through diffusion system through porcine skin (**a**). For the low detections of permeated compound, (**b**) is enhanced to show values (% ± SE with n replicates).

To track where the compounds partitioned during the experiment, measurements were taken from the swabs, tape, and skin. In Figure 2, Tape 1–3 is defined as the first three cellophane tape strips used to remove the top layers of skin while Tape 4–6 is defined as the last three cellophane tape strips used. Inner skin is defined as the 0.64 cm^2^ area that was dosed while the outer skin is defined as the peripheral skin in the cell but not over the dosed area.

A majority of the dosed compounds either stayed in the solvent or were retained in the upper layers of skin. Approximately 46–58% of PFCAs dosed with artificial perspirant remained in the dosing vehicle. PFCAs seemed to prefer to stay in the sweat solution rather than permeate through skin. For the acetone vehicle, a majority of the detected PFCA compounds were either on top of or in the upper layers of skin. Both PFOA and PFBA had 18% and 30% retained on the upper skin but were removed with the soap solution. PFHxA had a majority in the first three tape strips of skin. PFCA was retained in the outer area of skin in the range 0.7–7.1% of total dose, while the inner skin range was 1.1–6.7%.

## 4. Discussion

Increasing molecular size and LogK_ow_ were found to be inversely proportional to the permeability coefficient for these three PFCA compounds, as shown by Ragnarsdottir et al. [13]. For molecular size, it is known that larger compounds are less likely to absorb through skin. However, LogK_ow_ is the partitioning of a compound between octanol and water and used to estimate the lipophilicity of a compound, and the higher the LogK_ow_, the more lipophilic a compound is expected to be. In dermal absorption studies, lipophilic compounds are expected to absorb through the lipid-rich stratum corneum better than lipophobic compounds. This study shows the reverse: with increasing LogK_ow_, there is decreasing absorption. The issue is PFCAs are both hydrophobic and lipophobic and do not correspond with LogK_ow_ to skin partitioning. Molecular size corresponds better to permeability coefficients for PFCAs.

The permeability coefficient for PFOA in both vehicles was comparable with what Franko et al. found at a similar dose, shown in Table 3 [10]. However, Ragnarsdottir et al. found the permeability coefficient of PFOA to be two orders of magnitude higher than what was found in this study [13]. This difference could be attributed to several factors such as occlusion, vehicle choice, membrane or use of a static cell. Occlusion typically results in increasing permeability, contrary to the results in this study. Additionally, flow-through diffusion provides better sink conditions and more closely simulates *in vivo* conditions than static cells; however, no significant difference has been found between the two [16]. The use of 3D-HSE is likely the reason for the higher permeability coefficient. 3D-HSE is an artificial skin tissue that attempts to model human skin, while porcine skin has been demonstrated to better correlate with human skin [14]. Additionally, the use of methanol as a dosing vehicle could also impact the results found by Ragnarsdottir et al. [13].

For dermal absorption, there was no significant difference between artificial perspirant and acetone for PFBA, PFHxA, or PFOA. For compound distribution in skin, there was an apparent solvent effect. The PFCAs in artificial perspirant were generally collected in the initial swab, while in acetone a majority of compound was collected in the soap swab or initial tape strips. Collecting with swabs and tape strips is not precise. Some compound is likely lost to the sides of the dosing chamber or is retained in the swab. However, the high abundance of compound collected on the swabs and initial tape strips shows that PFCAs remained on or in the stratum corneum.

After the 8 h exposure, PFCAs appeared to be retained on the skin, which has been shown in several studies [3,11,13]. The focus of this study was on occupational exposure with an 8 h duration while most other studies had durations of at least 24 h. While only 0.05–0.25% of the applied dose was detected in the permeate, some of the retained PFAS could continue to permeate through the system after the exposure. The use of acetone resulted in PFOA and PFBA being retained in the skin at a higher concentration than with artificial perspirant. If more compound is retained in the skin, it can act as a reservoir resulting in an increased duration of permeation.

Recovery totals should be approximately 100%; however, these results ranged from 44% to 69%, shown in Figure 2a, which is considerably lower. One reason for the potential loss of PFAS could be from transfer. PFAS prefer the walls of plastic and glass containers rather than aqueous solvents [17]. Some PFAS may have been retained in the wall of the diffusion cell, flow-through tubing, or in the glass scintillation vials. However, this is likely a small amount. Another potential avenue for this loss was the collection of auxiliary sources such as swabbing the skin. With flow-through recovery comparisons, generally the focus is on what was collected in the flow-through with the auxiliaries to explain what is left over. In this study, a majority of the detected PFCAs were in either swabs or the top layers of skin with tape strips. There was likely loss due to extraction of the swabs and tape not being sufficient to absorb the large amount of dose left on the skin. Additionally, some of the chemical could have been transferred to the diffusion cell walls or retained in the swab rather than dispersing into the solvent. Since the low recovery was likely from the auxiliary collection, the absorbed dose and permeability coefficient would not be impacted. Future *in vitro* studies with PFAS should consider using methanol extraction to help ensure all PFAS is removed from scintillation vials.

## 5. Conclusions

This study shows that porcine skin is a sufficient model for measuring the permeability of PFOA in human skin. We observed a permeability coefficient for PFOA that correlated well with that found by Franko et al. [10]. While more data are needed to correlate the absorption of PFAS in porcine skin to that in human skin, future dermal studies should consider porcine skin when human skin is not available. The vehicle was not found to impact permeability; however, higher levels of compound were retained in the skin using acetone. Future studies should consider using artificial perspirant to provide conditions closer to *in vivo*.

This study found that PFOA, PFHxA, and PFBA do permeate skin within an 8 h duration. This information could help to evaluate the health risks associated with occupational exposure to PFAS. Occupational workers are exposed to PFAS for short durations but consistently due to wearing PFAS-treated gear. Dermal absorption of PFAS is a potential route but more information is needed on how much is bioaccessible from gear. Residual PFAS can remain on the skin surface from wearing PFAS-treated gear, so washing skin afterwards could reduce exposure.

PFCAs are only a subclass of PFAS and do not represent all PFAS. Studies are needed to measure the dermal permeability of volatile PFAS such as 6:2 fluorotelomer methacrylate, which has been found at high levels on firefighter turnout gear [18]. Previous studies have estimated that dermal absorption is a significant route of exposure for volatile PFAS [8]. *In vitro* studies are needed to assess the risk of volatile PFAS.

## Figures and Tables

**Table 1:** PFCA Compounds sourced from American Radiolabeled Chemicals.

Compound	Formula	Structure	Molecular Weight (g/mol)	Specific Activity (mCi/mmol)	Concentration (mCi/mL)	Water Solubility (mol/L) [15]	LogK_ow_ [15]
Perfluorobutanoic acid (PFBA)	C_4_HF_7_O_2_	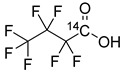	216.03	50	0.1	1.38 × 10^−2^	2.6
Perfluorohexanoic acid (PFHxA)	C_6_HF_11_O_2_	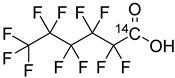	316.05	50	0.1	1.70 × 10^−3^	4.27
Perfluorooctanoic acid (PFOA)	C_8_HF_15_O_2_	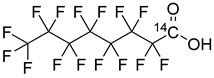	416.06	50	0.1	8.55 × 10^−3^	5.68

**Table 2:** Permeability coefficients of PFCAs.

	K_p_ (cm/h)	
Compound	Artificial Perspirant	Acetone
PFOA	8.28 × 10^−0.5^ ± 8.06 × 10^−0.6^	5.73 × 10^−0.5^ ± 7.80 × 10^−0.6^
PFHxA	1.31 × 10^−0.4^ ± 2.20 × 10^−0.5^	1.72 × 10^−0.4^ ± 3.34 × 10^−0.5^
PFBA	2.70 × 10^−0.4^ ± 1.30 × 10^−0.4^	2.54 × 10^−0.4^ ± 5.43 × 10^−0.5^

**Table 3:** Comparison of permeation variables for studies on PFCAs.

	Current Study	Current Study	Current Study	Current Study	Ragnarsdottir et al. 2024 [13]	Current Study	Current Study	Franko et al. 2011 [10]	Franko et al. 2011 [10]	Franko et al. 2011 [10]	Ragnarsdottir et al. 2024 [13]
Compounds	PFBA		PFHxA			PFOA					
Vehicle	Acetone	Artificial Perspirant	Acetone	Artificial Perspirant	Methanol	Acetone	Artificial Perspirant	Water	Citrate acid buffer	Water	Methanol
Permeability Coefficient (cm/h)	2.54 × 10^−4^	2.70 × 10^−4^	1.72 × 10^−4^	1.31 × 10^−4^	1.80 × 10^−2^	8.28 × 10^−5^	5.73 × 10^−5^	5.8 × 10^−5^	4.4 × 10^−5^	5.5 × 10^−2^	3.82 × 10^−3^
Dose Amount (µg/cm^2^)	16.3	17.8	11.3	12.8	0.5	21.3	15.8	10.9	2343.7	2343.7	0.5
Number of Replicates	5	6	8	8	3	6	7	8	10	8	3
Membrane	Porcine Skin	Porcine Skin	Porcine Skin	Porcine Skin	3D-HSE	Porcine Skin	Porcine Skin	Human Skin	Human Skin	Human Skin	3D-HSE
System	*In vitro*, flow-through	*In vitro*, flow-through	*In vitro*, flow-through	*In vitro*, flow-through	*In vitro*, static cell	*In vitro*, flow-through	*In vitro*, flow-through	*In vitro*, flow-through	*In vitro*, flow-through	*In vitro*, flow-through	*In vitro*, static cell
Occlusion	Occluded	Occluded	Occluded	Occluded	Unoccluded	Occluded	Occluded	Occluded	Occluded	Occluded	Unoccluded
Exposure Duration	8 h	8 h	8 h	8 h	36 h	8 h	8 h	24 h	24 h	24 h	36 h
Collection Duration	8 h	8 h	8 h	8 h	36 h	8 h	8 h	24 h	24 h	24 h	36 h

## Data Availability

The raw data supporting the conclusions of this article will be made available by the authors on request.

## Appendix A

**Table A1:** Compounds in Collection Media.

Compound	Amount
Sodium chloride	13.78 g
Potassium chloride	0.71 g
Calcium chloride	0.56 g
Potassium phosphate monobasic	0.32 g
Magnesium sulfate	0.58 g
Sodium bicarbonate	5.50 g
Dextrose	2.40 g
Bovine serum albumin	90.0 g
Distilled water	2 L
Sodium heparin (1000 units/mL)	10 mL
Amikacin (250 mg/mL)	0.25 mL
Penicillin G sodium (250,000 units/mL)	0.10 mL
